# Reply: Management of metastatic breast cancer: are we prepared to cope with our own success?

**DOI:** 10.1038/sj.bjc.6602295

**Published:** 2004-12-14

**Authors:** E Remák

**Affiliations:** 1 MEDTAP International, 20 Bloomsbury Square, London WC1A 2NS, UK

**Sir**,

We would like to thank the authors of the above letter for providing more recent information than our own. Our treatment pattern survey was carried out in 2001. It aimed to provide no more than a snap-shot of treatments used and treatment costs, and identify the trends that may lead to further cost increases. All three points noted by Jimeno and co-workers were discussed in our article as potential factors driving costs even higher.

Firstly, as discussed by Jimeno *et al*, the difference in incidence of initially metastatic breast cancer (IMBC) may be explained by the different data sources of the two studies. Our incidence figures were based on information from cancer registries, but registry databases do not include all patients with cancer, nor do they have complete staging information for every registered patient.

Secondly, survival with IMBC is highly variable, ranging from a few months to several years. Several studies indicate that survival is influenced by the sites of metastasis. Other factors such as time of diagnosis, availability of treatments and local treatment patterns may also have an impact as well as chance. The Royal Marsden Hospital data set used in our analysis contains information on over 2300 female patients with metastatic breast cancer *vs* the 370 patients in the study by Jimeno and co-workers, and was deemed a very reliable data source.

Lastly, treatment patterns change across countries and through time. As mentioned before, our resource use survey provided a snap-shot of treatments used in the UK in 2001. Since then, use of trastuzumab, the taxanes and aromotase inhibitors increased, and the costs are likely to increase further as their role in therapy continues to grow.

We agree with Jimeno and co-workers that metastatic breast cancer imposes a substantial economic burden, and this burden is likely to increase as the relatively more expensive treatments become more widely available.

